# What Role Do Different Types of Social Support Play in Life Satisfaction Among Korean Older Adults in Retirement?

**DOI:** 10.1093/geroni/igaa057.096

**Published:** 2020-12-16

**Authors:** Jihee Woo, Hyojin Choi

**Affiliations:** 1 University of Pittsburgh, Pittsburgh, Pennsylvania, United States; 2 Sungkyunkwan University, Seoul, Republic of Korea

## Abstract

Individuals are taking more responsibility for their retirement. However, economic stress generated by inadequate planning may pose a grave threat to well-being in retirement. Drawing on stress theory, this study examined how different types of social support mediate the relationship between economic stress and life satisfaction. We used the data from the 2013 and 2014 Korean Retirement and Income Study. Our sample was restricted to older Korean adults in retirement 55 to 96 years of age who were head of household (N=1,672). Confirmatory factor analysis was used to evaluate the measurement model for six latent constructs: 1) economic stress; 2) emotional support; 3) informational support; 4) instrumental support; 5) appraisal support; 6) life satisfaction. Structural equation modeling (SEM) was used to test this hypothesized model. The results revealed that both measurement model (CFI=0.985, TLI=0.983, RMSEA=0.036, SRMR=0.035) and structural model (CFI=0.978, TLI=0.984, RMSEA=0.030, SRMR=0.039) fit the data well. Standardized results from the SEM model adjusting for sociodemographic variables showed that economic stress directly predicted life satisfaction (β=-0.39, p<0.001) and two of four social support types directly predicted life satisfaction (instrumental support β=0.29, p<0.001; appraisal support β=0.25, p=0.004). Analyses demonstrated that economic stress may lead to lower levels of life satisfaction directly and indirectly through its effect on instrumental (β=-0.07, p<0.001) and appraisal support (β=-0.04, p=0.013). These findings will help inform policymakers and institutions of the need to alleviate economic stress and increase particular types of support with potentially more serious impact on the well-being of older adults in retirement.

